# Premature Aging in Skeletal Muscle Lacking Serum Response Factor

**DOI:** 10.1371/journal.pone.0003910

**Published:** 2008-12-11

**Authors:** Charlotte Lahoute, Athanassia Sotiropoulos, Marilyne Favier, Isabelle Guillet-Deniau, Claude Charvet, Arnaud Ferry, Gillian Butler-Browne, Daniel Metzger, David Tuil, Dominique Daegelen

**Affiliations:** 1 Institut Cochin, Université Paris Descartes, CNRS (UMR 8104), Paris, France; 2 Inserm, U567, Paris, France; 3 INRA, UR1282 Infectiologie Animale et Santé Publique, Nouzilly, France; 4 UMR S787, Inserm/UPMC-Paris 6/ Institut de Myologie, Paris, France; 5 Université Paris Descartes, Paris, France; 6 IGBMC (Institut de Génétique et de Biologie Moléculaire et Cellulaire), Department of Functional Genomics, Inserm, U596, CNRS, UMR 7104, Collège de France, Illkirch, Université Louis Pasteur, Strasbourg, France; Hospital Vall d'Hebron, Spain

## Abstract

Aging is associated with a progressive loss of muscle mass, increased adiposity and fibrosis that leads to sarcopenia. At the molecular level, muscle aging is known to alter the expression of a variety of genes but very little is known about the molecular effectors involved. SRF (Serum Response Factor) is a crucial transcription factor for muscle-specific gene expression and for post-natal skeletal muscle growth. To assess its role in adult skeletal muscle physiology, we developed a post-mitotic myofiber-specific and tamoxifen-inducible SRF knockout model. Five months after SRF loss, no obvious muscle phenotype was observed suggesting that SRF is not crucial for myofiber maintenance. However, mutant mice progressively developed IIB myofiber-specific atrophy accompanied by a metabolic switch towards a more oxidative phenotype, muscular lipid accumulation, sarcomere disorganization and fibrosis. After injury, mutant muscles exhibited an altered regeneration process, showing smaller regenerated fibers and persistent fibrosis. All of these features are strongly reminiscent of abnormalities encountered in aging skeletal muscle. Interestingly, we also observed an important age associated decrease in SRF expression in mice and human muscles. Altogether, these results suggest that a naturally occurring SRF down-regulation precedes and contributes to the muscle aging process. Indeed, triggering SRF loss in the muscles of mutant mice results in an accelerated aging process.

## Introduction

Skeletal muscle accounts for about 40% of the mass of the adult human body and is essential for maintaining and moving the skeleton, for breathing and for thermoregulation. Skeletal muscle is also one of the most metabolically active tissues in the body. The effects of aging on skeletal muscle include gradual loss of muscle mass, decline in muscle quality and functional properties. The decline in muscle quality is characterized by the presence of intramuscular fat, tissue fibrosis and a decreased regenerative potential. These age-related modifications in skeletal muscle, which have also been called sarcopenia, can be particularly severe and can affect both quality of life and lifespan of patients [1]. In order to counteract these modifications both in muscle structure and function, it is essential to understand the mechanisms involved in the maintenance of muscle mass and quality.

The regulation of muscle mass depends on a fine balance between protein synthesis and protein degradation which are controlled by different growth factors and signalling pathways [2]. For instance, activation of PI3Kinase/Akt/mTOR signalling downstream of IGF-1 increases protein content whereas activation of the ubiquitin-proteasome pathways through Atrogin1 and Murf1 stimulates protein degradation [3]. Whilst the mechanisms involved in skeletal muscle homeostasis have been rather well described, those involved in the age-related decline in muscle quality have not yet been fully elucidated. Age-related wasting and decreased regeneration potential of muscles have been associated to the decline in satellite cell function with age rather than to their number. Components of the micro-environment, or “niche”, which supports satellite cell activation, may vary with age. These components include: molecular signals expressed or secreted by the myofiber, basal lamina structure, products secreted by local interstitial cells (such as connective tissue, micro-vasculature, neural and immune system cellular components) and systemic factors [4]. Indeed, recent data has demonstrated the importance of endocrine/paracrine factors and Wnt pathways in age-related changes in muscle [5] as well as in more general aging processes [6].

At the molecular level, muscle aging is known to alter the expression of a variety of genes within the myofibers but very little is known about their molecular effectors [7]–[9]. Our recent findings have emphasized the role played by Serum Response Factor (SRF) in controlling post-natal muscle growth and transcription of locally expressed growth factors that are important for satellite cell accretion to growing myofibers [10]. SRF is a member of the MADS family of transcription factors that is produced in particularly large amounts in all muscles. *Via* binding to CArG box regulatory elements, SRF controls several extracellular stimuli-regulated genes important for cell growth, survival and migration, as well as muscle-specific genes. Moreover, several recent data -including ours- have demonstrated that SRF plays a key role in regulating the expression of many genes encoding muscle cytoskeletal and sarcomeric proteins including those for actins [10], [11]. SRF driven transcription of specific gene promoters can be regulated by different mechanisms, including modulating the level of SRF expression, SRF phosphorylation, nature and number of CArG boxes in the target gene, RhoA-mediated alterations in the cytoskeleton and association of SRF with different context or cell-specific co-factors. Several data suggest that SRF could be important when muscle mechanical loading is perturbed [12], [13]. For example SRF transcriptional activity is up-regulated by biomechanical stimuli *via* pathways involving integrin signalling [14]. SRF could thus play a central role in modulating gene expression to adapt muscle contractility to physiological demands.

In order to investigate SRF function in skeletal muscle growth and physiology, we created a model of targeted invalidation of SRF using the Cre-LoxP system [10]. However, the early and progressive evolution of the muscle phenotype, the fragility and the high degree of lethality of these mutant mice made it difficult to investigate the function of SRF in fully mature muscle physiology. By crossing mice homozygous for *SRF* floxed alleles (*S^f^/S^f^*) with *HSA-Cre-ER^T2^* transgenic mice [15] that express a tamoxifen (TAM) dependent Cre recombinase, exclusively in post-mitotic myofibers, we obtained a mouse model in which it is now possible to trigger at will the onset of SRF ablation in skeletal myofibers.

Here we show that mutant mice in which SRF is selectively ablated in adult post-mitotic myofibers develop a broad spectrum of alterations similar to those which have been described in age-advanced skeletal muscles. Thus, the age-related decrease in SRF that we observed in both mice and human muscles might contribute to muscle aging.

## Materials and Methods

### Generation of Mutant Mice

Mice homozygous for *SRF* floxed alleles [16] (abbreviated to *S^f^/S^f^*) and *HSA-Cre-ER^T2^* transgenic mice [15] have been described elsewhere. These two mouse strains were backcrossed onto a C57BL/6N genetic background for 7 generations prior to experimental analysis and then crossed to generate double-transgenic mice *HSA-Cre-ER^T2^:S^f^/+*. Subsequent breeding of *HSA-Cre-ER^T2^:S^f^/+* and *S^f^/S^f^* mice generated *HSA-Cre-ER^T2^:S^f^/S^f^* pre-mutant mice. Mice were genotyped by PCR, using DNA extracted from tail biopsies. In all experiments, groups of 2 months old male pre-mutant (*HSA-Cre-ER^T2^:S^f^/S^f^*) and sex- and aged-matched control (*S^f^/S^f^*) mice were given daily intraperitoneal TAM (1 mg per day; Sigma) injections for 5 consecutive days. Cre-mediated excision of SRF floxed alleles was detected as previously described [16]. All studies were conducted in accordance with European guidelines for the care and use of laboratory animals and were approved by the institutional animal care and use committee.

### Human Skeletal Muscle Tissues

Muscle biopsies were obtained via the french “Banque de Tissue pour la Recherche de l'AFM” (BTR-AFM) according to the French legislation on ethical rules. In general this material was surgical waste from orthopedic surgery. All biopsies had received patient consent and were anonymous. Samples were snap frozen in liquid nitrogen and stored at −80°C until use.

### Induction of Cardiotoxin-Induced Muscle Injury

Muscle regeneration was induced as described previously [17]. In brief, TAM-injected control and mutant mice were anaesthetised with Isoflurane (0.75 to 1.0% in oxygen) and received a single injection of cardiotoxin (CTX) (50 µl at 12 µM, intramuscular injection, Latoxan) into the Tibialis Anterior (TA) muscle. After 21 days *in vivo* muscle strength was measured the mice were killed, the TA muscle was removed and subsequently processed for histological analysis.

### Measurement of *in vivo* Muscle Strength

*In vivo* muscle strength was measured in order to evaluate the recovery from injury. Twenty-one days after muscle injury, animals were anaesthetised (pentobarbital, 60 mg/kg) and the *in situ* isometric contractile properties of the injured TA muscles were studied. In order to do this the distal tendon was attached to an isometric transducer (Harvard Bioscience, Les Ulis, France) using a silk ligature. The body temperature was maintained at about 37°C by means of heating lamps. The nerves innervating the muscles (proximally crushed) were stimulated with a bipolar silver electrode using a supramaximal square wave pulse of 0.1 ms duration. Measurements were made at L0 (length at which maximal tension was obtained during the twitch). Maximal tetanic force (P0; stimulation frequency of 75–143 Hz) was recorded and analysed on a personal microcomputer, using PowerLab system and Chart 4 software (ADInstruments, Paris, France). Once the contractile measurements had been made, the animals were killed with an overdose of pentobarbital. Muscles were then weighed, frozen in liquid nitrogen respectively precooled in isopentane and stored at −80°C until histological analyses were carried out.

### Muscle Histology, Immunohistochemistry, and Morphometric Measurements

All experiments involved the evaluation of control and mutant littermates and were conducted on at least 5 mice per group. Hindlimb muscles from 2 to 15 months old mice were removed and embedded in Tragacanth Gum, frozen in isopentane cooled in liquid nitrogen, and sectioned in a microtome cryostat (Leica). For the assessment of tissue morphology or visualization of fibrosis, 7-µm-thick transverse sections of the TA muscles were stained respectively with hematoxylin and eosin (H&E) and Sirius red and examined under a light microscope. Lipid accumulation was detected in the TA muscle fibers using Oil Red-O staining following fixation in 3% paraformaldehyde according to Koopman *et al.* [18], nuclei were visualised by counterstaining with DAPI (Molecular Probes). For fiber type analysis, serial sections of the plantaris muscle were processed either with a set of antibodies against the various myosin heavy chain (MyHC) isoforms, as previously described [19] or stained for the presence of SDH enzyme activity. Fiber area was analysed by incubating plantaris muscle sections with mouse anti-dystrophin Dys2 antibody (Novocastra) and staining them with Hoechst [20]. Between 600 and 1000 myofibers were analyzed per muscle. For each muscle, the distribution of fiber cross-sectional area (CSA) was determined by using Metamorph, version 2.56, software. For SRF immunodetection, paraffin-embedded longitudinal TA muscle sections were incubated with a 1∶200 dilution of a rabbit polyclonal antibody directed against the carboxy-terminal domain of SRF (G-20, Santa Cruz Biotechnology), as described elsewhere [16]. TA sections were also stained with modified Gomori trichrome to reveal tubular aggregates as red spots inside fibers in the “superficial” region of muscle. For their quantification, 700 fibers per muscle were analysed (n = 3 muscles for control and mutant) and their frequency was determined by plotting the number of tubular aggregates per individual fiber versus the percentage of fibers with tubular aggregates.

Images were taken using a Nikon Statif Eclipse E600 microscope with 20× or 40× magnification, 1.4-0.7 NA PL-APO objectives, a DXM1200 cooled CCD camera (Nikon) and ACT-1 (Universal Imaging).

### Electron Microscopy

Electron microscopy was performed on Plantaris muscles from mutant and control adult mice 13 months after TAM injection, as previously described [21].

### Western Blot Analysis

Western blotting was performed on various mouse muscles and human muscle biopsies as previously described [16]. Membranes were blotted overnight at 4°C with anti-SRF G-20 (1∶200), anti-glyceraldehyde-3-phosphate dehydrogenase (anti-GAPDH; 1∶2000) (Santa Cruz Biotechnology) and anti α-Tubulin (1∶2000) antibodies (Sigma). The precursor and mature forms of Sterol Regulatory Element Binding Protein-1c (SREBP-1c) were detected using a monoclonal antibody raised against the N-terminus (amino acids 301–407) of human SREBP-1 (NeoMarkers), and Fatty Acid Synthase polyclonal antibody (FAS) was a gift from I. Dugail (INSERM U465, France). After washes in TBS-T, membranes were incubated with horseradish peroxidase-conjugated goat anti-mouse or anti-rabbit secondary antibody (Pierce, Rockford, USA) and then revealed using the enhanced chemiluminescence system (Supersignal; Pierce). Signals were scanned and quantified using a ChemiGenius Apparatus (Syngene).

### Quantitative RT-PCR Analysis

Total RNA was extracted from Gastrocnemius muscles using a commercial kit (RNeasy, Qiagen) following the manufacturer's protocol. cDNA was synthesized from 2 µg of RNA using Moloney Murine Leukemia Virus reverse transcriptase (Invitrogen) and random hexamers (Promega). Quantitative PCR analysis was performed using a Light Cycler (Roche) according to the manufacturer's instructions using a SYBR Green I kit (Roche). Briefly, the final volume of 10 µl contained 16 ng of reverse-transcribed total RNA, 10 µM of primer mix, 6 µl of the Reaction Mix SYBR Green. Reactions were carried out in capillaries (Roche) with 40 cycles. The comparative threshold cycle (CT) method was used to calculate the relative concentrations. This method involved obtaining CT values for the transcripts of interest normalizing to the housekeeping gene *Cyclophilin* and comparing the relative differences between control and mutant samples. Primer sequences are available on request.

### Statistical Analysis

Results are expressed as means±standard errors of the means (SEM). The significance of differences between means was assessed with Student's t test. P values of <0.05 were considered statistically significant.

## Results

### Efficient Tamoxifen-Induced Loss of SRF in Skeletal Muscle

**Figure 1:**
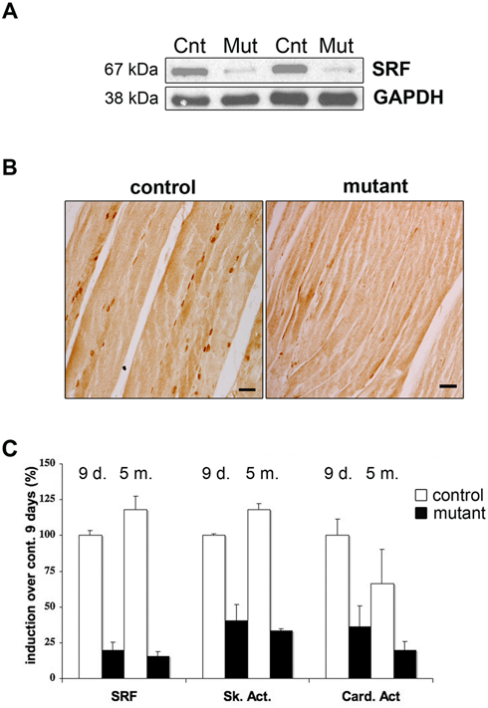
Validation of the skeletal muscle-specific SRF KO model after TAM injection. (A) Immunoblot analysis of protein extracts from control (Cnt) and mutant (Mut) muscles 9 days after TAM injection using an anti-SRF antibody. Anti-GAPDH is used as a loading control. (B) Immunostaining of longitudinal sections from control and mutant TA muscles with the anti-SRF antibody 9 days after TAM injection. Bars: 50 µm. (C) Quantitative real-time PCR was performed on RNAs prepared from gastrocnemius muscles of control (white) and mutant (black) mice 9 days (9 d.) and 5 months (5 m.) after TAM injection (n = 4 for controls and mutants at each time). Mean expression levels for *SRF*, *Skeletal* and *Cardiac α-actin* mRNAs were normalized using *Cyclophilin* transcript as a reference.

*HSA-Cre-ER^T2^:S^f^/S^f^* pre-mutant mice were healthy and fertile for months without any sign of muscle alteration. Two months old male *HSA-Cre-ER^T2^:S^f^/S^f^* pre-mutant mice and *S^f^/S^f^* control littermates were TAM-injected, and hereafter are referred to as mutants and controls, respectively. Following TAM injection, Cre-mediated excision of *S^f^* floxed alleles was found to be restricted to skeletal muscle (data not shown) and within nine days after the first injection resulted in an 80% down regulation of the *SRF* mRNA level in mutant muscles (Fig 1C). The decrease in *SRF* levels was found to be similar in different muscles. Consistent with these findings, western blotting showed that SRF protein levels were 80% lower in mutant compared to control skeletal muscles (Fig. 1A) and in mutants the majority of the myonuclei were no longer immunostained for SRF (Fig 1B). The efficient loss of SRF-dependent gene expression in mutant muscles was also confirmed by a concomitant and major reduction in the mRNA levels of known SRF target genes, such as *skeletal* (60%) and *cardiac* (60%) *α-actin* (Figure 1C) as well as *muscle creatine kinase* to a lesser extent (data not shown). More importantly, the loss of expression of both *SRF* and *actin* persisted with time, as illustrated by the analysis of the mutant muscles 5 months after TAM-injection (Fig 1C).

### Development of Atrophy and Fibrosis in Mutant Muscles of Adult Mice

**Figure 2:**
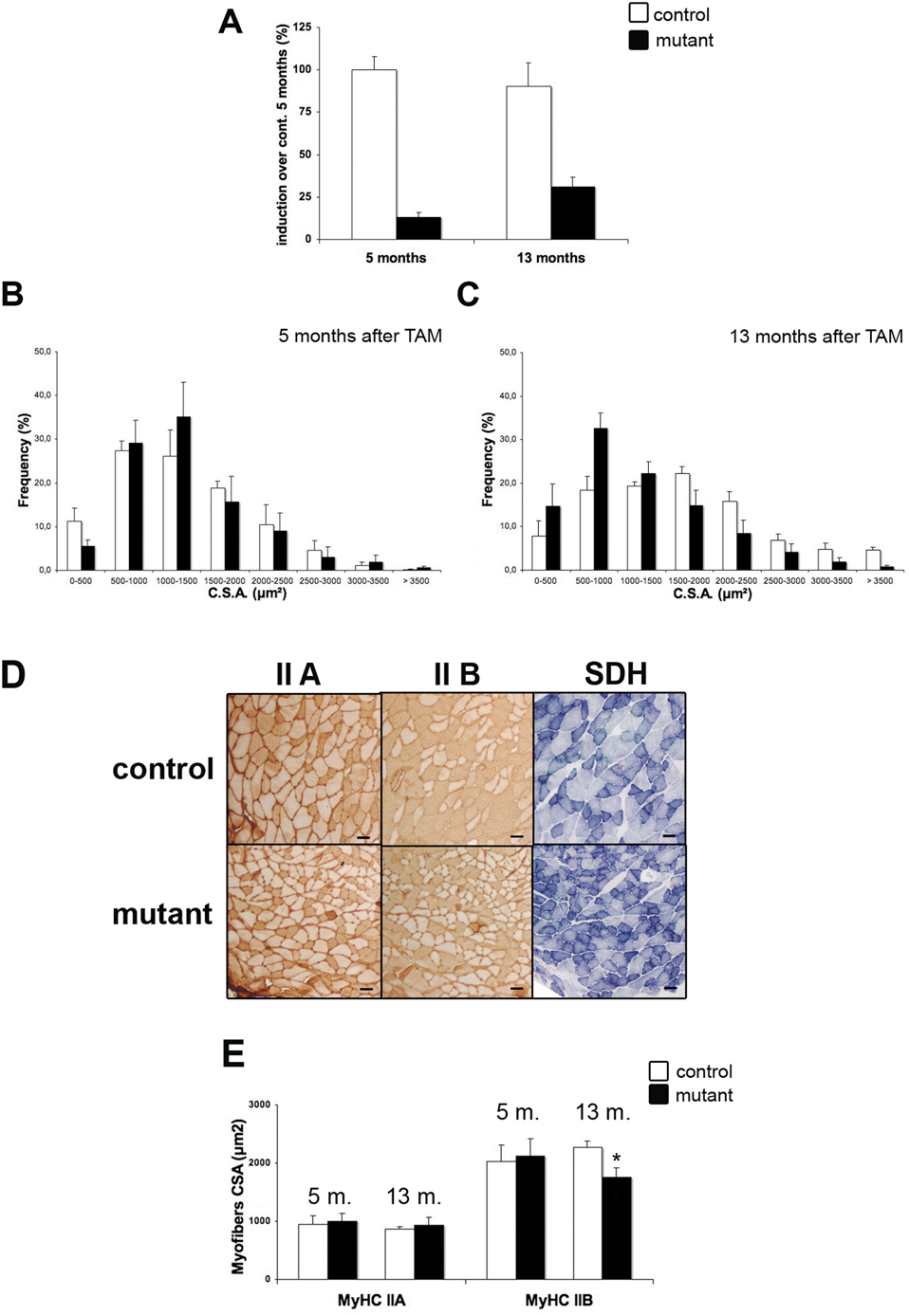
Progressive atrophy of MyHC-IIB fibers and metabolic switch in SRF deficient muscles. (A) Quantitative real-time PCR was performed on RNAs prepared from gastrocnemius muscles of control (white) and mutant (black) mice 5 months (5 m.) and 13 months (13 m.) after TAM injection (n = 4). Mean expression levels for *SRF* mRNAs was normalized using *Cyclophilin* transcripts as a reference. (B, C) Frequency histogram showing distributions of muscle fiber CSA in control (white) and mutant (black) plantaris muscles 5 (B) and 13 (C) months after TAM injection (n = 4 for control and mutant at each time; 600 fibers were analysed for each muscle) (D) Immunostaining analysis using MyHC-IIA,-IIB antibody (left and middle panels) and SDH staining (right panel) of transversal sections of control and mutant plantaris muscles 13 months after TAM injection. Bars: 50 µm. (E) Mean myofiber CSA of MyHC-IIA or -IIB expressing myofibers from control (white) and mutant (black) 5 and 13 months after TAM injection (n = 4, 600 myofibers were measured for each muscle). *P<0.05 over controls 13 months after TAM injection.

**Figure 3:**
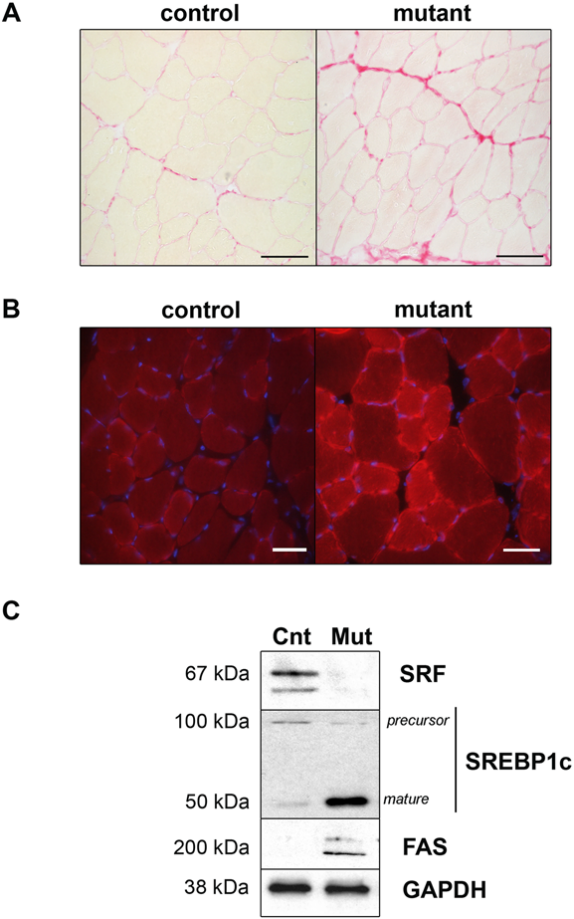
Fibrosis accumulation and alteration of lipid metabolism in SRF deficient muscles. (A) Sirius Red staining of transversal sections of control and mutant TA muscles 13 months after TAM injection. Bars: 50 µm. (B) Oil Red O staining of transversal sections of control and mutant TA muscles 13 months after TAM injection. Nuclei are counter-stained with DAPI. Bars: 50 µm. (C) Immunoblot analysis of protein extracts from control (Cnt) and mutant (Mut) muscles 8 months after TAM injection using anti-SRF, anti-SREBP1c and anti-FAS antibodies. Anti-GAPDH is used as a loading control.

In spite of an important down-regulation of *skeletal* and *cardiac α-actin* transcripts and in the absence of any detectable compensation by other actin isoforms, such as *smooth muscle α-actin*, γ *and β actins* (data not shown), the mutant mice remained healthy several months after TAM injection when kept in standard conditions. When analyzed 5 months after TAM injection (7 months old mice), no obvious difference could be observed between controls and mutants in body weight, in myofiber CSA or in oxidative/glycolytic muscle fiber type patterning, as illustrated in table 1. However, 13 months after TAM injection (15 months old mice), mutant muscles displayed signs of atrophy. At this time, *SRF* expression was still 60% lower in mutant muscles, indicating a limited contribution of the recruitment of new nuclei derived from satellite cells, which express SRF, to the adult myofibers (Fig 2A). As illustrated in Fig 2B, no difference was observed in myofiber CSA distribution between controls and mutants 5 months after TAM injection (Fig. 2B). In contrast, 13 months after TAM injection, the fiber size distribution was altered in mutants, with the vast majority of the mutant muscle fibers having a CSA between 500 and 1000 µm2, whereas the mean myofibers CSA for control muscle fibers was between 1500 and 2000 µm2 (Fig. 2C). The CSA of the muscle fibers depends on their contractile and energy metabolism status. In the plantaris muscle which is mainly composed of fibers that express MyHC-IIA and -IIB (80%), fast/oxidative type IIA fibers have a smaller CSA than fast/glycolytic IIB fibers. Therefore, we investigated whether the distribution of oxidative/glycolytic myofiber subtypes was modified in mutant muscles, using antibodies specific to MyHC-IIA and -IIB isoforms. No significant differences were observed 5 months after TAM injection (table 1). In contrast, 13 months after TAM injection, immunostaining showed that the mutant Plantaris muscle contained fewer MyHC-IIB myofibers (38% versus 54% in control) and more MyHC-IIA myofibers (43% versus 27% in control) as illustrated in Fig. 2D and Table 1. SDH staining was performed in order to assess whether the MyHC fiber type modification was accompanied by a metabolic switch from glycolytic to oxidative metabolism. In agreement with MyHC typing data, we observed an increase in the number of myofibers with high SDH staining in muscles lacking SRF since 13 months (Fig. 2D; Table 1). Moreover, measurement of the mean CSA of the various myofiber subtypes revealed a specific atrophy of MyHC-IIB fibers in mutant muscle (Fig. 2E). Hence, both a specific MyHC-IIB fiber atrophy and a switch towards a more oxidative phenotype seemed to account for the atrophy observed in mutant muscles. This phenotype is strongly reminiscent of what is observed in aged muscles, with IIB myofibers being more prone to age related muscle atrophy [22]. Another classical sign of skeletal muscle aging is an increase in tissue fibrosis [5]. We therefore investigated the presence of fibrosis by Sirius red staining in 15 months old control and mutant mice. As illustrated in Fig. 3A, mutant muscles exhibited endomysial fibrosis, whereas such an accumulation of fibrotic tissue has never been observed in control muscles at the same age.

**Table 1:** Plantaris muscle characteristics from control and mutant mice 5 and 13 months after TAM injection.

	7-months-old		15-months-old	
	Controls	Mutants	Controls	Mutants
**% MyHC IIA**	35±5,4	37±1,2	27,5±10,2	44±3,2 *
**% MyHC IIB**	43±4,6	38±6,8	54±9,9	38±5,3 *
**% SDH+**	42±2,2	44,5±2,2	30,4±5,3	55,2±6,9 *
**Body weight (g)**	22,5±1,9	20±0,6	33,4±3,6	32,3±3,6

* P<0,05 over controls 13 months after TAM injection.

### Fat Accumulation in Mutant Muscles

Age-related changes in skeletal muscle are also characterised by an increased fat content [1]. We investigated a possible alteration in the lipid content of mutant muscles in comparison to aged-matched control muscles by using Oil Red O staining that highlights the accumulation of neutral lipids. As illustrated in Figure 3B, Oil Red-O positive droplets were abundant in 15 months old mutant muscles, whereas only a weak staining was observed in age-matched control muscles (Fig. 3B). Muscles lacking SRF accumulated both extracellular and intramyocellular lipids. We have previously shown that intramyocellular lipids resulted mainly from *de novo* lipogenesis (lipid synthesis from glucose) in rodent muscle fibers [23]. To determine whether intramyocelullar lipid accumulation resulted from lipogenesis in mutant muscle, we measured the protein levels of two actors of lipogenesis, the Sterol Regulatory Element Binding Protein-1c (SREBP-1c), a key lipogenic transcription factor that has been shown to regulate the transcription of genes involved in fatty acid metabolism, and *Fatty Acid Synthase* (*FAS*), one of the SREBP-1c targets. At the molecular level, lipid accumulation was shown 8 months after TAM injection (10 months old mice) in mutant muscles to be correlated with an up-regulation of the transcriptionnally active form of SREBP-1c protein (Fig. 3C). This was correlated with an up-regulation of FAS in the mutant muscles, whereas no increase was observed in TAM-injected age-matched controls. The increase in SREBP-1c expression was not accompanied with an up-regulation of *SREBP-1c* mRNA, thus excluding a direct transcriptional control by SRF (data not shown). These results suggest that lipogenesis is activated in mutant muscles and can already be detected 8 months after TAM injection, which is not the case in age-matched control muscles.

### Altered Sarcomere Ultrastructure in Mutant Muscles

**Figure 4:**
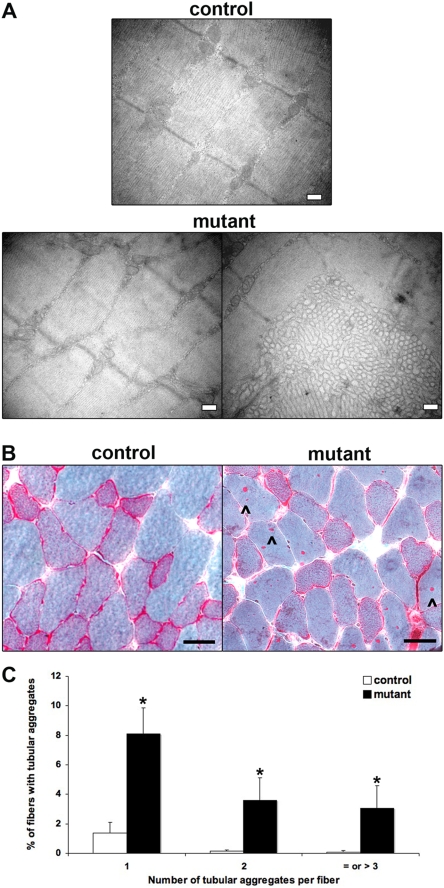
Impaired sarcomere ultrastructure in SRF deficient muscles. (A) Representative electron micrographs of longitudinal sections of control and mutant Plantaris muscles. Well-structured sarcomere units are observed in control myofibers 13 months after TAM injection. Major changes to the ultrastructure in the male mutant Plantaris muscle are illustrated by sarcomere unit disorganisation (left panel) with the presence of tubular aggregates (right panel). Bars: 0.2 µm. (B) Modified Gomori trichrome staining of transversal sections of control and mutant Tibialis muscles 13 months after TAM injection. Arrowhead indicates the presence of tubular aggregates in the mutant muscle. Bars: 50 µm. (C) Quantification of the frequency of tubular aggregate formation in control (white) and mutant (black) TA muscles 13 months after TAM injection (n = 3 for control and mutant; 700 fibers were analysed for each muscle). The frequency was determined by plotting the number of tubular aggregates per individual fiber versus the percentage of fibers with tubular aggregates. *P<0.05 over control.

Since *skeletal* and *cardiac α-actin* transcription was found to be profoundly decreased in mutant muscles, we investigated the consequences of SRF loss on muscle structure. Ultrastructural analysis revealed changes in the structure of the 15 months old mutant myofibers. Whereas age-matched control muscle displayed regularly oriented actin/myosin filaments and well structured Z-disks, mutant muscles displayed sarcomere unit disorganisation (Fig. 4A, left panel). In addition, muscles of male mutant mice exhibited tubular aggregates that were detected 8 months after TAM injection (data not shown) and increased both in number and size 13 months after TAM injection (Fig. 4A, right panel). These tubular aggregates appeared as accumulations of densely packed tubules arising from sarcoplasmic reticulum. The accumulation of such aggregates has been described to be an age-related phenomenon in skeletal muscle of male mice [24]. To quantify these tubular aggregates, skeletal muscle sections from 15 months old control and mutant male mice (13 months after TAM injection) were stained with modified Gomori's trichrome that reveals tubular aggregates as red spots inside fibers (Fig. 4B). Figure 4C shows the distribution of the number of tubular aggregates per fiber in 15 months old control and mutant muscles. Only 1,6% of the fibers contained aggregates in control muscle, whereas 15% of mutant fibers contained aggregates and half of these had two or more aggregates per fiber.

These ultrastructural alterations combined with the myofibers atrophy were associated with a tendency towards a decrease in muscle strength 13 months after TAM injection. However, the individual variability and the small number of mice available for these studies did not allow us to observe a statistically significant reduction in strength but the mutants all displayed a lower maximal tetanic force by comparison with age-matched control (not shown).

### Impaired Regeneration in Mutant Myofibers

**Figure 5:**
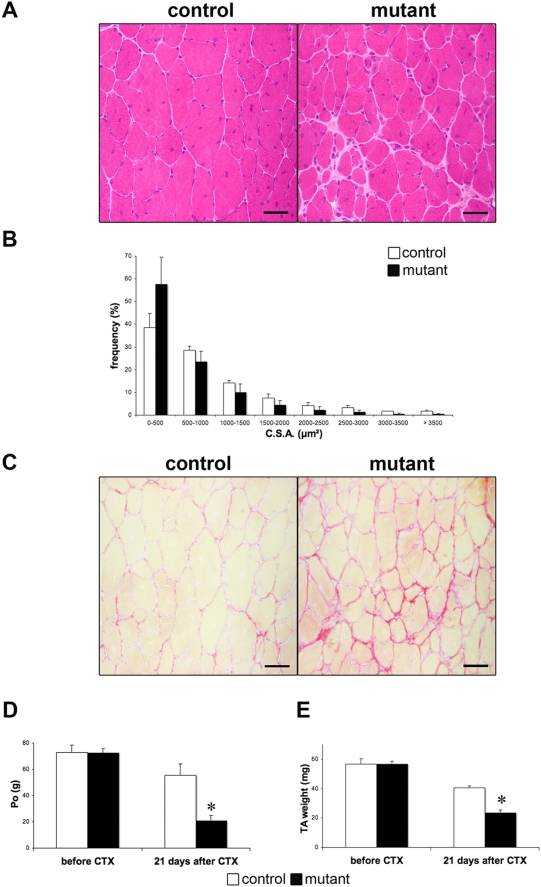
Impaired regeneration in SRF deficient muscles. (A) Representative H&E-stained of transversal sections of TA muscles from control and mutant mice 21 days after CTX injection. Bars: 50 µm. (B) Frequency histogram showing distributions of myofibers CSA in control (white) and mutant (black) regenerated TA muscles 21 days after CTX injection (n = 5; 1000 myofibers were analysed for each muscle). (C) Sirius Red staining of transversal section of control and mutant TA muscle 21 days after CTX injection. Bars: 50 µm. (D, E) Maximal tetanic force, PO (D) and TA weight (E) were measured before and 21 days after CTX injection from control (white) and mutants (black) mice. (n = 5) *P<0.05 over control 21 days after CTX.

To evaluate the role of SRF in muscle regeneration, we compared the muscle histological characteristics of 4 months old mutant and control mice after CTX injury. Muscle regeneration involves satellite cell activation and recruitment for fusion with damaged myofibers or for the production of new muscle fibers. As satellite cells of mutant muscles do not express the *Cre-ER^T2^* recombinase, control and mutant mice were injected every three days with TAM during the regeneration process to avoid possible re-expression of SRF in myofibers after satellite cell recruitment. Before regeneration, analysis of H&E-stained sections demonstrated no visible differences between the two types of mice regarding the histology of TA muscle (data not shown). Twenty-one days after injury, while a complete regeneration was observed in control muscles, characterized by centrally located, nuclei mutant muscles displayed increased interstitial spaces and smaller regenerated myofibers (Fig. 5A and B). Such altered regeneration was confirmed by comparing the regenerated myofiber CSA at day 21 post-injury. As presented in Fig. 5B, the size distribution of regenerated myofibers CSA of mutants is decreased as compared with regenerated control myofibers. To evaluate whether the enlarged interstitial spaces observed in the injured muscle from mutant mice were associated with the accumulation of fibrotic tissue, we performed Sirius Red staining. Regenerated mutant muscle displayed an important increase in fibrosis (Fig. 5C) that persists at least 3 months after CTX injury (not shown). To evaluate whether histological differences between the regenerated muscles from control and mutant mice were associated with a difference in the functional recovery from injury, maximal tetanic force was measured before and 21 days after CTX (Fig. 5D). After CTX injury, recovery of strength was 80% complete in control but only 20% in mutant mice. Maximal force production was related to muscle weight. Therefore, muscle mass was also measured. The mass of injured TA was also reduced by 40% in mutant mice compared with controls (Fig. 5E).

### Evolution of SRF Expression as a Function of Age in Mouse and Human Skeletal Muscle

**Figure 6:**
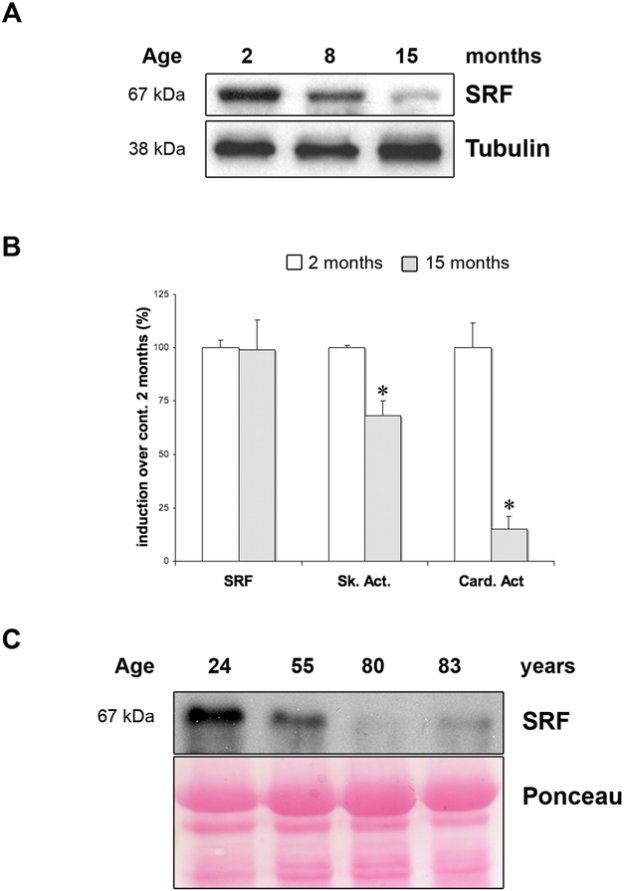
Age-dependant down-regulation of SRF expression in skeletal muscle of mice and human. (A) Immunoblot analysis of protein extracts from 2, 10 and 15 month-old control muscles using anti-SRF antibody. Anti-α-Tubulin is used as a loading control. (B) Quantitative real-time PCR was performed on RNAs prepared from Gastrocnemius muscles of control mice 2 (white) and 15 (grey) months old. Mean expression levels for *SRF*, *Skeletal* and *Cardiac α-actin* mRNAs was normalized using *Cyclophilin* transcript as reference. (n = 4). *P<0.05 over control 2 month-old. (C) Immunoblot analysis of protein extracts from human skeletal muscle (24, 55, 80 and 83 years old) using anti-SRF antibody. Ponceau staining is used as a loading control.

The overall phenotype of mutant mice at steady state and during regeneration is strongly reminiscent of age-related skeletal muscle abnormalities. These results prompted us to analyse the expression of SRF in skeletal muscles isolated from control mice at various ages. A progressive and important decrease in SRF protein expression, up to a 70% down-regulation at 15 months of age, could be observed in skeletal muscle with age (Figure 6A). This SRF down-regulation did not occur at the transcriptional level as illustrated by Real Time PCR (Fig. 6B, left). However, the functionality of SRF was impaired as confirmed by a 30% down-regulation of *skeletal α-actin* transcripts and an 80% down-regulation of *cardiac α-actin* transcripts with age in 15 months old control muscles (Fig. 6B). In humans as in mice, skeletal muscle regeneration is impaired with age [25] and there is an increase in fat within and between the muscle fibers [1]. We thus wanted to know whether SRF was also down-regulated in the skeletal muscle of elderly humans. Western blots of muscle biopsies from humans at different ages showed that whereas SRF protein was abundantly expressed in skeletal muscles of young subjects (24 years old) as the age of the subjects increased (80 and 83 years old) we observed a progressive decrease in the expression of SRF (Figure 6C).

## Discussion

In the present study, we have investigated the role of SRF in the physiology of adult skeletal muscle by using an inducible Cre-mediated *SRF* gene disruption strategy that allows us to trigger the onset of SRF loss only within mature post-mitotic myofibers [15].

In contrast to the drastic phenotype observed in previous skeletal muscle-specific SRF KO models affecting growing myofibers [10], [26], inducing SRF loss in adult myofibers did not produce an overt phenotype within the first months after triggering *SRF* disruption. While our data show that SRF remains a major regulator of *skeletal* and *cardiac α-actin* transcription in the context of adult skeletal muscle, they also suggest that SRF is much less crucial for the maintenance of fully differentiated myofibers than for pre and peri-natal muscle growth. However, most notably, we show here that with time, mutant skeletal muscles develop a wide spectrum of alterations including atrophy, fibrosis, lipid accumulation and a perturbed regeneration. In combination, all these features are characteristic of aged skeletal muscle [1], suggesting that triggering SRF loss in adult myofibers leads to a premature aging process. Most interestingly, we also observed decreased SRF levels in the skeletal muscle of aged mice and humans, suggesting that a naturally occurring decrease in SRF expression could contribute to the muscle phenotype observed during the aging process and in sarcopenia.

### An Inducible Model of SRF Ablation that Mimics an Accelerated Skeletal Muscle Aging

By using Cre transgenes with different temporal expression patterns during muscle development, our laboratory and others have observed that SRF may have different functions towards its known target genes according to the developmental stage of the skeletal muscle [10], [26]. Such differences in SRF transcriptional activity have also been observed in embryonic versus adult heart [16], [27]. Since SRF seems to be able to shift between different gene expression programs according to the physiological state, of the muscle. The ability to inactivate SRF at a specific and controlled time point may help us to understand its different specific functions. TAM-dependent activation of the *Cre-ER^T2^* led to an efficient, but not complete deletion of the *SRF* gene in skeletal muscle fibers, as revealed by the presence of a small proportion of immunostained nuclei for SRF in mutant myofibers. This was confirmed by the fact that, about 20% of *SRF* transcripts were detected by quantitative RT-PCR in TAM-injected mutants whereas, using the same approach, very few *SRF* transcripts could be detected in our previous constitutive muscle-specific SRF KO model in which the Cre was driven by the same human *skeletal α-actin* promoter (data not shown) [10]. Such residual amounts of SRF might be sufficient to activate a certain sub-set of SRF-dependent genes but not others. This could explain why the transcription of the *skeletal α-actin* gene that contains 4 CArG boxes and is a major SRF target [28] is down-regulated by 60%, whereas the transcription of the IGF-1 gene with one active CArG box [10] was not affected in this model (data not shown). Another possibility is that SRF is needed for *IGF-1* transcription in the context of the growing myofiber, but not in the adult. A number of studies have shown that there is an age-related decline in the level of muscle IGF-1 during sarcopenia. Aging muscle has also been associated with an attenuation of the ability of exercise to induce IGF-1 [29]. Moreover, an over-expression of the *mIGF1* isoform in mouse muscle has been shown to protect against age-related muscle alterations [30]. In contrast, other data shows that there is no local down-regulation of *IGF-1* mRNA in aged muscle and that, the most sarcopenic individuals showed higher local expression of *IGF-1* mRNA [31]. Accordingly, in our model, we can exclude the implication of a loss of *IGF-1* expression within the myofibers in the accelerated aging process that is observed. Our data demonstrate that this inducible model of SRF ablation mimics what occurs naturally and progressively during normal mouse muscle aging. Moreover, we show that once an 80% SRF loss has been induced in 2 months old mice, the installation of the phenotype is very progressive. The phenotype begins to be observed at 8 months after TAM injection (lipid accumulation, tubular aggregates) but all of the alterations, associated with premature aging, can only be clearly observed at 13 months after TAM injections. In 15 months old control muscles, in spite of a significant and natural decrease in SRF protein (70%) and a concomitant down-regulation of the target *actin* genes, we could not observe a particular muscle phenotype. Similar but milder muscle alterations to those observed in mutant mice could only be observed in very old mice (25 months old; data not shown). Altogether these data strongly suggest that the natural and progressive decrease of SRF could contribute to some of the age-related muscle alterations and that our model of inducible SRF down expression reproduce an accelerated aging process.

### Mutant Muscles Develop a Late Fiber-Type Switch and Selective IIB-Myofibers Atrophy

In steady state conditions, the residual levels of *SRF* and *actin* transcripts seem to be sufficient for the maintenance of normal mature muscle fibers for at least 5 months after TAM injection. However, with time the loss of SRF resulted in a global myofiber atrophy as evidenced by the alteration of the fiber size distribution. Since oxidative myofibers have a smaller CSA, this decrease in fiber size may be due at least in part to the overall switch towards more oxidative fibers that we observed in mutant muscles. This switch in fiber types is associated with an increase of the IIA/IIB fiber ratio which is also in line with an increased oxidative metabolism. This result is in accordance with what we previously observed in the non-inducible muscle-specific SRF KO [10] and data from the literature showing that SRF activates the fast/glycolytic fiber-specific MyHC IIB gene promoter and represses the fast/oxidative fiber-specific MyHC IIA promoter [32]. Dy/dy dystrophic mice that have reduced muscle SRF content [33] also exhibit decreased MyHC IIB levels [34], [35]. Although we cannot exclude that the slow/oxidative phenotype of mutants could be related to a physiological adaptation of muscles to the premature aging, our data support a role for SRF in maintaining the expression of the *MyHC IIB* gene.

Mutant muscles also develop a selective atrophy of fast MyHC IIB expressing myofibers. Interestingly, it has been reported that the age-related decrease in skeletal muscle mass observed in humans is also caused by a selective atrophy of type II fibres [22]. As the starting SRF level is lower in fast/glycolytic muscles than in slow/oxidative ones [13], we propose that the down-regulation of SRF in IIB myofibers through normal aging or TAM-induced in mutant mice have earlier impact than in other myofibers types, thus leading to specific atrophy.

The decreased expression of the *skeletal* and *cardiac α-actin* that occurred in mutant muscle and to a lesser extent in aging muscles could also contribute to the atrophy and to the alterations in sarcomere organization that are observed in mutants. Skeletal α-actin comprises about 90% of the muscle actin in adult mouse skeletal muscle and corresponding knockout mice die during the perinatal period from abnormalities in skeletal muscle growth and force generation [36]. In mutant muscles, the sarcomeric disorganisation was associated with the presence of tubular aggregates that are another sign of muscle senescence.

### A Possible Role for SRF in Controlling Muscle Lipid Content

The phenotype of sarcopenia includes a loss of muscle mass with an increase in intramuscular fat and connective tissue [22], [37]. In muscles triggered for SRF loss, we observed an important lipid accumulation. We could show that this increased muscle lipid content is due at least in part to *de novo* lipogenesis as the mature form of SREBP1-c is up-regulated and its target gene *FAS* is activated. Most interestingly, several data obtained in different cellular models have already pointed to an inverse correlation between SRF activity and the activation of an adipogenic program. Indeed, the nuclear localisation of SRF is repressed during both senescence and adipocyte differentiation of murine 3T3 mesenchymal stem cells [38]. The anticancer drug distamycin A, which induces the adipocyte differentiation of 10T1/2 pluripotential cells, selectively inhibits the binding of SRF to DNA [39]. Mechanical stretch, that is known to activate SRF expression [13], was shown to inhibit C2C12 myoblast-to-adipocyte differentiation [40]. Altogether, our *in vivo* results strongly support an active role for SRF in inhibiting an “adipogenic” program within myofibers. When SRF loss is triggered, such an inhibition would be alleviated, leading to the up-regulation of the mature form of SREBP1-c. However, SRF does not act directly on *SREBP1-c* transcription, as we did not observe any up-regulation of *SREBP1-c* transcripts in mutant muscles. The mechanisms linking SRF to the inhibition of lipid synthesis remains to be investigated.

### Role of SRF in Regeneration and Fibrosis

The regenerative potential of skeletal muscle declines with age and this impairment is associated with an increase in tissue fibrosis [5]. In the previous non-inducible muscle-specific SRF KO, we observed that the mutant muscle fails to regenerate. In this model, before injury, muscle lacking SRF presented a myopathy characterized by a drastic myofiber hypotrophy and impaired maturation [10]. In the new model of inducible SRF loss described here, we show that, 2 months after TAM injection, whereas mutant myofibers lacking SRF appear normal, the regeneration process is also altered, with the presence of smaller regenerated myofibers, persistent endomysial fibrosis and reduced strength. These results emphasize a direct role for SRF during regeneration. In this model, as in the previous one, satellite cells are not targeted by the SRF disruption and their intrinsic properties are preserved [10]. Thus, alterations in the growth of regenerated muscle fibers in mutant mice most probably stems from a modified satellite cell niche. Such modification could result from an altered myofiber signalling or environment coming from mutant myofibers. The important fibrosis observed in mutant muscles either with aging in mutant muscles or after regeneration suggests that the lack of SRF in myofibers also contributes to modify the environment in a way favouring the appearance and the maintenance of fibrosis. Consistent with this finding, myofibers from transgenic mice expressing a dominant negative form of the MRTF-A SRF co-factor that suppresses SRF activity, also displayed extensive fibrosis [26].

The critical role of age-related changes in the satellite cell niche and the resulting impairment of muscle regeneration has been recently underlined [4]. In this scope, the myofiber itself constitutes one of the main niche components and our model in which it is the only mutated component strongly supports a major role for alterations coming from the myofibers in the age-related process. The particular role of SRF within the myofiber for maintaining a proper satellite cell environment and/or profile of secreted myokines remains to be characterized [41].

### Modulation of SRF Expression with Aging

The phenotype of premature aging observed in muscles triggered for SRF loss demonstrates that the significant decrease in the amount of SRF protein that we observed with time in control skeletal muscles could at least in part contribute to the aged-muscle phenotype. Importantly, we could show that SRF expression is also impaired with aging in humans. In accordance with our results, very recently, Sakuma *et al.* reported a decrease in both SRF and MRTF-A with aging, in the skeletal muscle of mice [42]. Moreover, the expression of SRF targets such as *actin* has been shown to be down-regulated during aging in rats [43] and humans (*cardiac α-actin* but also *egr1* and *c-fos*) [7]. Thus, the maintenance of SRF expression seems to be critical to ensure a proper response of skeletal muscle to damaging insults and to prevent age-related abnormalities. Interestingly, the fine tuning of SRF expression and activity has also been shown to be very important for the function of the cardiac and smooth muscle tissues. In these tissues, age-related alterations in SRF levels were also associated to pathological states. Hence, in contrast to what we observed in skeletal muscle, SRF expression is increased in the hearts of old rats and mice in the basal conditions [44], [45]. This age-related increase correlates with a cardiac pathogenic phenotype that is alleviated when SRF activity is down-regulated through the expression of a mutant SRF protein [44]. Consistent with these finding, *cardiac α-actin* expression is up-regulated in old myocardium [46]. An increased expression of SRF and cognate target genes in vascular smooth muscle cells seems to account for the arterial hypercontratility observed in Alzheimer disease [47]. Hence the modulation of SRF expression, function and localisation seems to be very important in maintaining the integrity of muscle tissues. The aging process would alter this tissue-specific fine tuning of SRF expression and activity, leading in turn to the progressive development of further muscle alterations and sarcopenia in human skeletal muscles. Resistance training is currently the most effective known strategy to combat sarcopenia. It is interesting to note that SRF plays a central role in transducing mechanical signals from cell membranes to skeletal fiber nuclei *via* pathways involving integrin β1, RhoA and Focal Adhesion kinase [14], [48]. Moreover, mechanical activity is known to increase SRF expression [12], [13].

Altogether, our data show that the model of inducible muscle-specific SRF KO that we describe here could represent a key genetic tool for studies designed to elucidate the mechanisms involved in impaired skeletal muscle maintenance in human aging.
